# Exploring Black Soldier Fly Frass as Novel Fertilizer for Improved Growth, Yield, and Nitrogen Use Efficiency of Maize Under Field Conditions

**DOI:** 10.3389/fpls.2020.574592

**Published:** 2020-09-23

**Authors:** Dennis Beesigamukama, Benson Mochoge, Nicholas K. Korir, Komi K. M. Fiaboe, Dorothy Nakimbugwe, Fathiya M. Khamis, Sevgan Subramanian, Thomas Dubois, Martha W. Musyoka, Sunday Ekesi, Segenet Kelemu, Chrysantus M. Tanga

**Affiliations:** ^1^International Centre of Insect Physiology and Ecology, Nairobi, Kenya; ^2^Department of Agricultural Science and Technology, Kenyatta University, Nairobi, Kenya; ^3^Department of Crop Production and Management, Busitema University, Tororo, Uganda; ^4^International Institute of Tropical Agriculture (IITA), Yaoundé, Cameroon; ^5^Department of Food Technology and Nutrition, School of Food Technology, Nutrition & Bioengineering, Makerere University, Kampala, Uganda

**Keywords:** agronomic nitrogen use efficiency, frass fertilizer, *Hermetia illucens*, maize yield, nitrogen fertilizer equivalence, nitrogen recovery efficiency, nitrogen uptake

## Abstract

Black soldier fly frass fertilizer (BSFFF) is increasingly gaining momentum worldwide as organic fertilizer. However, research on its performance on crop production remains largely unknown. Here, we evaluate the comparative performance of BSFFF and commercial organic fertilizer (SAFI) on maize (H513) production. Both fertilizers were applied at the rates of 0, 2.5, 5, and 7.5 t ha^-1^, and 0, 30, 60, and 100 kg nitrogen (N) ha^-1^. Mineral fertilizer (urea) was also applied at 0, 30, 60 and 100 kg N ha^-1^ to establish the N fertilizer equivalence (NFE) of the organic fertilizers. Maize grown in plots treated with BSFFF had the tallest plants and highest chlorophyll concentrations. Plots treated with 7.5 t ha^-1^ of BSFFF had 14% higher grain yields than plots treated with a similar rate of SAFI. There was a 27% and 7% increase in grain yields in plots treated with 100 kg N ha^-1^ of BSFFF compared to those treated with equivalent rates of SAFI and urea fertilizers, respectively. Application of BSFFF at 7.5 t ha^-1^ significantly increased N uptake by up to 23% compared to the equivalent rate of SAFI. Likewise, application of BSFFF at 100 kg N ha^-1^ increased maize N uptake by 76% and 29% compared to SAFI and urea, respectively. Maize treated with BSFFF at 2.5 t ha^-1^ and 30 kg N ha^-1^ had higher nitrogen recovery efficiencies compared to equivalent rates of SAFI. The agronomic N use efficiency (AE_N_) of maize treated with 2.5 t ha^-1^ of BSFFF was 2.4 times higher than the value achieved using an equivalent rate of SAFI. Also, the AE_N_ of maize grown using 30 kg N ha^-1^ was 27% and 116% higher than the values obtained using equivalent rates of SAFI and urea fertilizers, respectively. The NFE of BSFFF (108%) was 2.5 times higher than that of SAFI. Application rates of 2.5 t ha^-1^ and 30 kg N ha^-1^ of BSFFF were found to be effective in improving maize yield, while double rates of SAFI were required. Our findings demonstrate that BSFFF is a promising and sustainable alternative to commercial fertilizers for increased maize production.

## Introduction

Improving food security in sub-Saharan Africa (SSA) requires strategies that can match future food demands with increasing population growth while conserving the soil resources. High levels of hunger and food insecurity dominate most regions of Africa, and the situation continues to worsen due to increasing soil degradation (FAO, 2017). Forty percent of soils in SSA are low in nutrient stocks, with 25% affected by aluminum toxicity, 18% prone to leaching and 8.5% characterized by phosphorus fixation (Tully et al., 2015). Most soils in SSA are deficient in nitrogen (N), phosphorus (P) and potassium (K) (Cobo et al., 2010) while most farm lands in East Africa have negative macronutrient balances (Gachimbi et al., 2005; Ebanyat et al., 2010) and yet, very little (≤ 10 kg ha^-1^) or no mineral fertilizer is used (FAO, 2017). Even in the few farms where mineral fertilizers are used, low nutrient use efficiencies and crop yields have been reported (Ebanyat et al., 2010; Kihara et al., 2016; Liverpool-Tasie et al., 2017), mostly attributed to low soil organic matter levels, many micronutrient deficiencies and high acidity (Vanlauwe et al., 2015; Musinguzi et al., 2016).

Like in many SSA countries, most soils in Kenya are low in organic matter, with levels even below the critical value of 3% (Gachimbi et al., 2005), and exhibit high acidity (Keino et al., 2015). This has led to high P fixation, making P a limiting factor to crop production. Furthermore, uptake and utilization of macronutrient (mostly N, P and K) is affected by the low availability of secondary nutrients [calcium (Ca), magnesium (Mg) and sulfur (S)] and most micronutrients in soils (Tittonell et al., 2008a; Wortmann et al., 2019). Despite this situation, most farmers do not apply organic matter, which could help to replenish some of the secondary nutrients and micronutrients into the soil through mineralization (Baligar et al., 2001; Grigatti et al., 2015; Ch’Ng et al., 2016).

Previous research efforts on soil fertility management in SSA (Tittonell et al., 2008b; Vanlauwe et al., 2014; Vanlauwe et al., 2015) have recommended combined application of mineral and organic fertilizers to improve and sustain soil fertility and crop yields. When applied across farms in Kenya, combined organic and mineral fertilizers have improved crop yields, nutrient use efficiencies and soil fertility (Mucheru-Muna et al., 2007; Mucheru-Muna et al., 2014; Musyoka et al., 2017). A major challenge hindering the use of organic fertilizers is the limited source of organic matter, since most organic resources have other competing uses such as feeding livestock on the farm (Rufino et al., 2011; Ndambi et al., 2019). Therefore, alternative sources of organic fertilizer for farm use such as insect frass are needed.

The increasing demand for animal feed through black soldier fly (*Hermetia illucens* L.) (BSF) mass rearing using organic waste (van Huis, 2013; Makkar et al., 2014) presents an avenue for organic waste management that could also contribute to soil fertility improvement. The BSF larvae are rich in proteins, fats and minerals (Caruso et al., 2014). The BSF larvae have a high waste degradation efficiency (65%–79%) (Diener et al., 2011) and can significantly reduce pathogens present in waste (Lalander et al., 2015). The frass, which is a byproduct from BSF rearing contains substantial amounts of nutrients (Lalander et al., 2015; Oonincx et al., 2015) that could be useful in crop production if converted into organic fertilizer. The frass fertilizer generated would also increase income from insect farming through the sale of organic fertilizer as a second product from BSF rearing or save the farmer from incurring fertilizer purchase costs.

The use of insect BSF frass as organic fertilizer is a relatively new concept. Adoption of a new concept or product as fertilizer in any farming system requires information on its performance in terms of how it influences crop growth, yield, nutrient uptake, and use efficiency in comparison to existing fertilizers. The effectiveness of organic fertilizers highly depends on the source, nutrient content, stage of mineralization, and storage method (Rufino et al., 2007; Ebanyat, 2009; Ndambi et al., 2019). For example, nutrient availability in manure is greatly influenced by the source, mineralization status and C/N ratio (Grigatti et al., 2015; Musyoka et al., 2019).

Most research efforts on use of insect frass as a fertilizer have been conducted under controlled conditions (Kagata and Ohgushi, 2012; Poveda et al., 2019; Houben et al., 2020). Those that have involved BSF frass (Choi and Hassanzadeh, 2019) have been performed under potted conditions, without assessing the economic yield and nutrient utilization. Most research outputs from greenhouse or potted experiments cannot be directly transferred to field phase without being tested due to variations in production environments. Information on the performance of BSF frass on crop performance under field conditions is lacking. Furthermore, the optimum application rates and comparative performance of BSF frass fertilizer in relation to existing organic fertilizers is not documented. In addition, the nitrogen fertilizer equivalence of BSF frass fertilizer—an important index that would provide information on the amount mineral N fertilizer saved while using N from BSF frass fertilizer to achieve the same crop yield—is largely unknown. Therefore, the aim of this study was to determine the performance of BSF frass fertilizer on nitrogen availability and uptake, growth, yield, and nitrogen use efficiency in maize, to generate information necessary for recommendation of BSF frass fertilizer into existing farming practices.

## Materials and Methods

### Study Site Characteristics

Field experiments were carried out for two seasons (April–September 2019 and October 2019–March 2020) at the Kenyatta University teaching and demonstration farm (1° 10′ 59″ S, 36° 55′ 34″ E) located in Nairobi County, Kenya at an elevation of 1580 m above sea level. Nairobi County receives bimodal rainfall with annual averages of 925 mm. The first rainfall season starts from March to June while the second season runs from October to December. The mean monthly temperatures of Nairobi ranges between 21°C and 28°C (www.meteo.go.ke).

**Figure 1:**
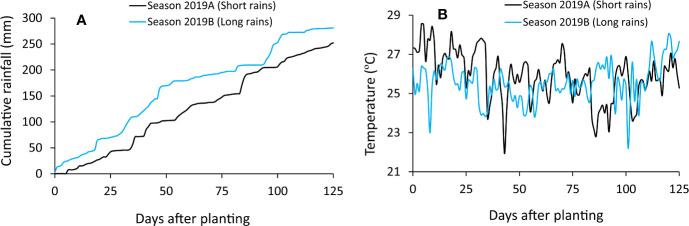
Cumulative rain fall **(A)** and mean daily temperatures **(B)** during field experiments.

During the experiments, daily temperature and rainfall data were sourced from Kenyatta University weather station, located about 0.5 km from the experimental site. Mean daily temperatures of 22°C–29°C and 22°C–28°C were recorded at the experimental site during the short and long rain season, respectively. Cumulative rainfall totals of 252 and 281 mm were received during the short and long rain season experiments, respectively (**Figure 1**). Soils in the study site are Acric Ferralsols (Gachene and Kimaru, 2003) characterized by shallow depths, low organic matter, and low pH levels. Before experiments, soil sampling (0–20 cm) was done for determination of total organic N, total organic C, available P, exchangeable cations (K, Ca and Mg), pH, electrical conductivity (EC) and soil texture using procedures described in (Okalebo et al., 2002). **Table 1** shows selected physical-chemical characteristics of the soils used in the experiment.

**Table 1:** Selected physical and chemical characteristics of the experimental soil, and organic fertilizers used during experiments.

Experimental soil																			
Parameter	pH(1:2.5 water)	Bulk density	Mineral N		Total N	TOC	SOM	AvailableP (ppm)	Exchangeable cations(cmol kg^-1^)						% sand		% clay	% silt	Texturalclass
		(g cm^-3^)	(mg kg^-1^)		(%)				K		Ca		Mg						
**Test value**	5.9	1.35	1.81		0.04	1.3	2.3	9.7	2.07		0.91		0.07		63		20.3	16.7	Sandy loam
**Organic fertilizers**																			
**Parameters**	Moisture (%)	pH		EC (mS cm^-1^)	Ammonium		Nitrate	Total C	Total N	Total P		Total K		Total Ca		Total Mg			C/N ratio
					(mg kg^-1^)			(%)											
**BSF frass**	30.1	7.7		2.7	74.4		1.39	35.2	2.1		1.16		0.17		0.19		0.16		16.8
**SAFI organic**	29.8	6.4		6.1	39.4		92.3	45.1	3.0		1.23		1.49		0.29		0.43		15.0

TOC, total organic carbon; SOM, soil organic matter; EC, electrical conductivity.

### Source of Fertilizers

The experiment involved three fertilizers: BSF frass fertilizer, commercial organic fertilizer (SAFI), and a mineral N fertilizer (45% urea). The BSF frass fertilizer was a product obtained from the feeding of BSF larvae on a substrate made of brewery spent grains (from Kenya Breweries limited) at the International Centre of Insect Physiology and Ecology (*icipe*, Nairobi, Kenya). The BSF larvae were reared according to Shumo et al. (2019). After harvesting of larvae at 2 weeks, the frass was composted for 5 weeks using the heap method to obtain a mature and stable frass product, which was used in experiments as BSF frass fertilizer. The commercial organic fertilizer was sourced from Safi organics limited (http://safiorganics.co.ke/) located in Mwea town, Kirinyaga County, Kenya. It was a mixture of chicken manure, biochar, and rock phosphate. **Table 1** shows selected physical-chemical characteristics of the organic fertilizers used in the experiments. Urea was sourced from Kenya Farmers’ Association stores, Nairobi.

### Treatments and Experimental Setup

The study consisted of two experiments, each repeated for two seasons. In the first experiment, the BSF frass fertilizer and SAFI were applied singly at 0, 2.5, 5.0, 7.5 t ha^-1^ to determine the most effective rate for crop production. These were denoted as control, 2.5BSF, 5BSF and 7.5BSF for BSF frass fertilizer, and 2.5SAFI, 5SAFI, and 7.5SAFI for SAFI treatments. Henceforth, throughout the text, tables and figures these treatments are referred to as “types of fertilizers”.

In the second experiment, the organic fertilizers were applied at four rates equivalent to 0, 30, 60 and 100 kg N ha^-1^ to determine the optimum N rate. These were denoted as control, 30N BSF, 60N BSF and 100N BSF for BSF frass fertilizer treatments, and 30N SAFI, 60N SAFI and 100N SAFI for SAFI treatments. The N rates of 30, 60, and 100 kg N ha^-1^ were equivalent to 1.4, 2.9, and 4.8 t ha^-1^ of BSF frass fertilizer, respectively, and 1.0, 2.0, and 3.3 t ha^-1^ of SAFI organic fertilizer, respectively. The mineral N fertilizer (45% N urea) was applied at equivalent rates (30, 60 and 100 kg N ha^-1^) as the organic fertilizers. In addition, the grain yields from the urea fertilizer treatment were used to draw the N response curve for determining the N fertilizer equivalencies (NFE) of organic resources.

To remove any limitation in the mineral fertilizer and organic fertilizers applied in terms of N rates, inorganic P [supplied as triple super phosphate – TSP (46% P_2_O_5_)] and K [supplied as muriate of potash (60% K_2_O)] were obtained from Kenya Farmers’ Association and applied at uniform rates of 60 kg P ha^-1^ and 50 kg K ha^-1^ (Tittonell et al., 2008a). For organic fertilizers treatments, inorganic P and K were applied as top up to the nutrients (P and K) already contained in the dry matter used to supply respective N rates. The second experiment, where fertilizers were applied in terms of N rates and supplemented with P and K is referred to as nitrogen fertilizer treatments in this document. The control treatment was not amended in both experiments.

The maize variety H513, which is recommended for low and medium altitude areas of Kenya, was used as the test crop. The experiments were set out as randomized complete block design (RCBD) with three replicates. Plots measured 4 × 4 m with border widths of 0.5 m and 1 m between the plots and blocks, respectively. The TSP fertilizer was applied at planting while urea and muriate of potash were applied in two splits: 50% at 4 weeks after planting and another 50% at 7 weeks after planting.

### Maize Growth, Yield, and Mineral N

Plant height and chlorophyll concentrations were measured from 10 plants per plot that were randomly selected and tagged for repeated measurements at early vegetative [35 days after planting (DAP)] (8–10 leaves), late vegetative (55 DAP) (13–16 leaves), tasseling (70 DAP) and silking stages (91 DAP). Plant heights were measured using a tape measure from the ground level up to the apex of the topmost leaf while chlorophyll concentration was measured using a SPAD meter placed on the fourth fully opened leaf from the top (Yuan et al., 2016). Biomass amounts and nitrogen uptake were determined at early vegetative, tasseling, silking and maturity (125 DAP) (harvesting) stages. Two plants were randomly selected from each plot, cut at ground level and their fresh weights determined. Thereafter, their subsamples were oven dried at 60°C for 72 h; after cooling the dry weights were determined. The dried samples were ground into powder which was used for the determination of total N.

Grain yield data was collected at harvesting period from each plot area after all the ears had dried. Plants in the harvested area were cut at ground level and their ears threshed to determine grain weights and weight of residues using a weighing scale. Grain and stover samples were taken to the laboratory and air-dried to 12.5% moisture content for determination of grain and residues yields per plot and on a hectare basis (t ha^-1^). Part of the grain and stover sample from each treatment was ground into powder for determination of N, P and K concentrations.

During plant sampling, soil samples were also collected at two depths (0–20 cm and 20–40 cm) to determine mineral N (nitrate-N and ammonium-N) concentration during crop growth. A soil auger was used to collect sub-samples from eight spots within the inner four rows of each plot. The subsamples were homogenized by the quarter sampling approach (Okalebo et al., 2002) to obtain representative samples. The soil samples were placed in air-tight polythene bags and carried to the laboratory using cool boxes containing ice blocks to reduce microbial activities during transportation.

### Nitrogen Use Efficiencies

The following N use efficiencies were calculated; the total N uptake by plants as measured in plant tissues and grain, agronomic efficiency, apparent N recovery efficiency, agro-physiological N efficiency, and N harvest index. Part of the air-dried samples were ground using an analytical mill for analysis of N in grain and plant tissues, which were used to measure the amount of N in grain and plant residues per treatment (equation 1). Agronomic efficiency (AE_N_), which is a measure of economic yield produced per unit N supplied from each treatment, was calculated using grain yield from each treatment (equation 2). Apparent N recovery efficiency (ANR_N_) was calculated to determine the ability of the plant to acquire the N supplied from different treatments (equation 3). Agro-physiological N efficiency (APE_N_) was calculated to determine the economic yield per unit N accumulated from each fertilizer treatment (equation 4). Nitrogen harvest index (NHI) was calculated to determine the fraction of nitrogen accumulated in the grain (equation 5). All the N use efficiency indices were calculated according to Baligar et al. (2001).

(1) $$N\;uptake\;(kg\;N\;ha^{-1})=\frac{\left[N(\%)\times dry\;matter(kg\;ha^{-1})\right]}{100}$$

(2) $$AE_{N}(kg\;kg\;N^{-1})=\frac{\left[Yield_{F}(kg\;ha^{-1})-Yield_{C}\;(kg\;ha^{-1})\right]}{Quantity\;of\;N\;applied\;(kg\;N\;ha^{-1})}$$

(3) $$ANR_{N}(\%)=\frac{\left[N\;uptake_{F}(kg\;N\;ha^{-1})-N\;uptake_{C}\;(kg\;N\;ha^{-1})\right]}{Quantity\;of\;N\;applied\;(kg\;N\;ha^{-1})}\times 100$$

(4) $$APE_{N}(kg\;kg\;N^{-1})=\frac{\left[Yield_{F}(kg\;ha^{-1})-Yield_{C}\;(kg\;ha^{-1})\right]}{\left[{\left(\begin{array}{c} N\;uptake\;in\\ grain\;and\;stover \end{array}\right)}_{F}kg\;N\;ha^{-1}-{\left(\begin{array}{c} N\;uptake\;in\\ grain\;and\;stover \end{array}\right)}_{C}kg\;N\;ha^{-1}\right]}$$

(5) $$NHI(\%)=\frac{N\;uptake\;in\;grain\;(kg\;N\;ha^{-1})}{N\;uptake\;in\;grain\;and\;stover\;(kg\;N\;ha^{-1})}\times 100$$

Where,

*F* represents plots that received fertilizer treatments,

*C* represents the control plots.

### Nitrogen Fertilizer Equivalence Values of BSF Frass and SAFI Fertilizers

**Figure 2:**
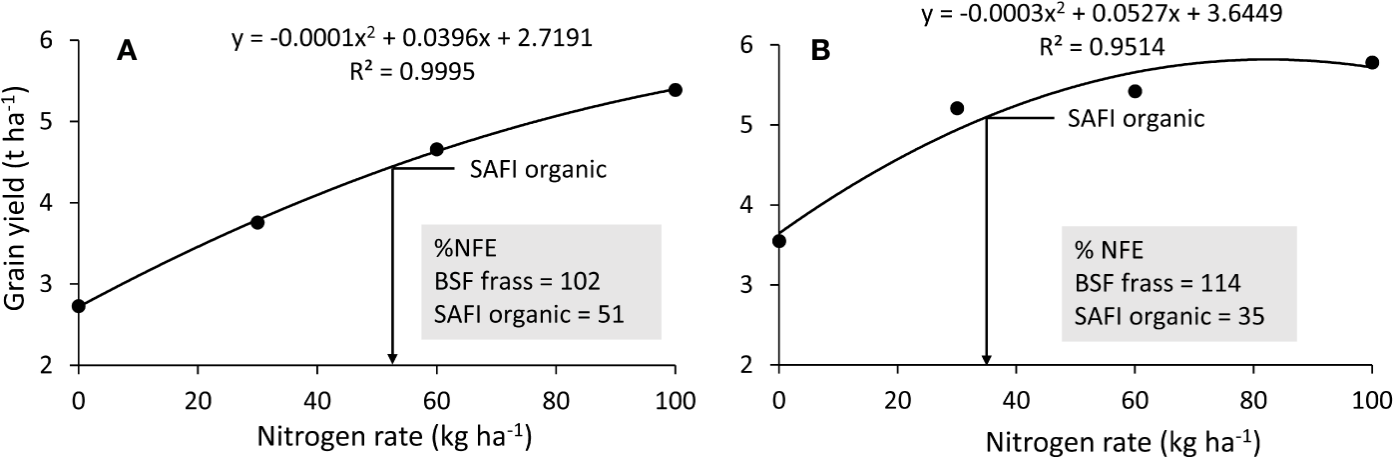
Maize grain yield response to curve used to determine fertilizer equivalence values of organic fertilizers applied at 100 kg N during short **(A)** and long rain season **(B)**. (Note: grain yields from black soldier fly (BSF) frass fertilizer were above N response curve in both seasons).

The values of grain yields from the urea fertilizer treatments (0, 30, 60, and 100 kg N ha^-1^) were used to draw the N response curves (**Figure 2**). Grain yields from organic fertilizers applied at 100 kg N ha^-1^ were used to calculate their nitrogen fertilizer equivalence (NFE) from the N response curves using equation 6 (Murwira et al., 2003). Nitrogen fertilizer equivalence is the amount of mineral N fertilizer saved when using organic fertilizer N to achieve the same crop yield (Hijbeek et al., 2018). Nitrogen fertilizer equivalence is expressed as kg kg^-1^ but is mostly converted into percent for comparison purposes (Kimetu et al., 2004; Cavalli et al., 2016; van Middelkoop and Holshof, 2017).

(6) $$NFE(\%)=\frac{\left[FR\;(kg\;ha^{-1})\times 100\right]}{N\;applied\;(kg\;ha^{-1})}$$

Where,

*FR* represents the fertilizer response of organic fertilizers obtained by comparing their grain yields with that of the N response curve.

Since the response assumed a quadratic function with the equation Y = aFR^2^+ bFR + c, the equations were solved using the quadratic formula. The constants a, b and c were obtained from the N response curves.

### Laboratory Analysis Methods

Total N in soil was determined using Kjeldahl digestion and distillation method, while soil texture was determined using Bouyoucos hydrometer method (Okalebo et al., 2002). The pH and EC were determined using extracts of 1:10 and 1:2.5 [weight/volume (w/v)] for organic fertilizer to distilled water and soil to distilled water, respectively. The contents were then shaken for 1 h at 180 revolutions min^-1^ on an orbital and linear shaker (MI0103002, Four E’s Scientific, China). The pH and EC were then read directly using a pH (AD1000, Adwa, Romania) and EC meter (AVI, Labtech, India), respectively (Okalebo et al., 2002).

The mineral N (nitrate and ammonium) was extracted from organic fertilizers and soil using 0.5 M potassium sulpfate at a ratio of 1:10 (w/v). Thereafter, the entire content of compost-potassium sulfate mixture was shaken for 1 h using an orbital and linear shaker (KOS – 3333/KCS – 3333, MRC, UK) as described above. The solution was filtered using Whatman No. 1 filter paper and the filtrate was used for further analyses. The nitrate and ammonium concentrations were determined by colorimetric methods at 419 and 655 nm, respectively, following procedures described by Okalebo et al. (2002). The mineral N concentration at each depth (0–20 cm and 20–40 cm) was converted to kg ha^-1^ using bulk density of each depth as determined at sampling period.

Total organic carbon of organic fertilizers and soil was determined using the wet oxidation method (Nelson and Sommers, 1982). The total N, P, K, Ca, and Mg of organic fertilizers were extracted using acid digestion (Okalebo et al., 2002). From this extract, total N, P, and K were determined using the Kjeldahl distillation method (Jackson, 1973), UV-Vis spectrophotometry method (Okalebo et al., 2002) and flame photometry (Okalebo et al., 2002), respectively, while Ca and Mg concentrations were determined using atomic absorption spectrometry (AAS) (iCE 3300 AA system, Thermo scientific, China) at 422.7 and 285.2 nm, respectively (Okalebo et al., 2002). Available P and exchangeable Ca and Mg in soil were determined using Bray 2 and AAS, respectively, while exchangeable K was determined using flame photometry.

### Data Analysis

Prior to analysis, data were checked for normality using the Shapiro-Wilk test. Plant height, chlorophyll concentration, soil mineral N concentration, and N uptake data were analyzed using a linear mixed-effect model with “lmer” function from the package “lme4” in R statistical software with fertilizer treatment and sampling time as fixed effects, and replication as random effect. Data on grain and stover yields, P and K accumulation in stover and grain, and nitrogen use efficiency were analyzed using one-way analysis of variance test. Computation of least squares means was done using “lsmeans” package, followed by mean separation using adjusted Tukey’s method implemented using “cld” function from the “multicompView” package. Data were analyzed separately for sole and N fertilizer treatments, and each season. All the statistical analyses were conducted using R software version 3.6.0 (R Core Team, 2019).

## Results

### Effects of BSF Frass, Commercial Organic, and Urea Fertilizers on Maize Plant Height

**Figure 3:**
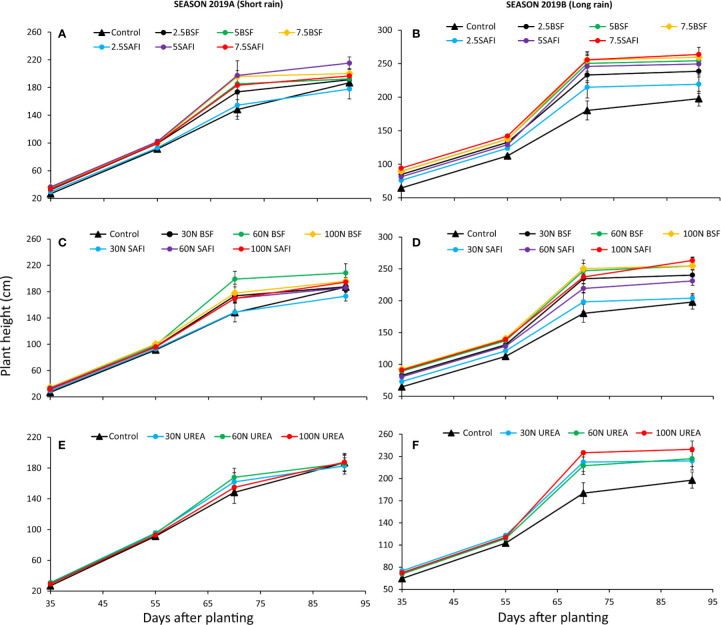
Effect of black soldier fly (BSF) frass fertilizer **(A–D)**, commercial organic fertilizer (SAFI) **(A–D)**, and commercial mineral fertilizer (urea) **(E, F)** on maize plant height.

The different types of fertilizers and N fertilizer rates showed significant differences in maize plant heights at different growth stages during the short (fertilizer types: p < 0.001, N fertilizer rates: p < 0.001) and the long rain seasons (fertilizer types: p < 0.001, N fertilizer rates: p < 0.001) (**Figure 3**). Plant heights significantly (p < 0.001) increased from early vegetative stage (27–94 cm) (35 DAP) to highest values (173–264 cm) at silking stage (91 DAP) with the smallest increases in plant heights observed at silking stage.

Among the different types of fertilizers, plots treated with 5 and 7.5 t ha^-1^ of SAFI produced the tallest plants during the short (215 cm) (p < 0.001) and long rain (264 cm) seasons, respectively (**Figures 3A, B**), but the long rain season was not significant (p = 0.26). For N fertilizer treatments, plots treated with organic fertilizers produced taller plants than urea treated plots (**Figures 3C–F**). Application of 60 kg N ha^-1^ supplied as BSF frass fertilizer produced the tallest plants (208 cm), that were 7% and 11% (p = 0.53) taller than those produced using equivalent rates of the commercial organic and urea fertilizers, respectively, during the short rain season. Plots treated with 100 kg N ha^-1^ supplied using SAFI produced the tallest plants (263 cm) during the long rain season, which were 3 and 10% (p < 0.001) taller than those grown using equivalent rates of BSF frass fertilizer and urea fertilizer, respectively. The control treatment and plots treated with 2.5 t ha^-1^ as well as 30 kg N ha^-1^ supplied using SAFI produced the shortest plants during the two seasons of the experiments.

### Effects of BSF Frass, Commercial Organic, and Urea Fertilizers on Leaf Chlorophyll Concentration

**Figure 4:**
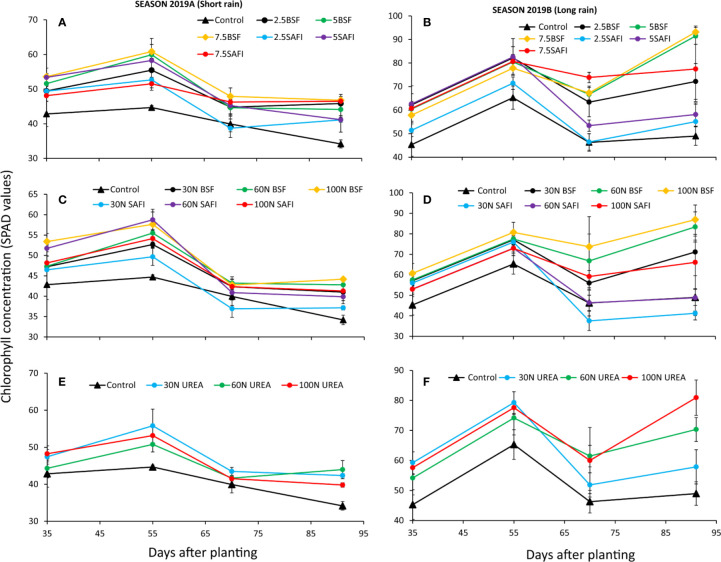
Effect of black soldier fly (BSF) frass fertilizer **(A–D)**, commercial organic fertilizer (SAFI) **(A–D)**, and commercial mineral fertilizer (urea) **(E, F)** on maize chlorophyll concentration.

The maize leaf chlorophyll concentration varied significantly at different growth stages due to different types of fertilizers during the short (p < 0.05) and long rain seasons (p < 0.01) (**Figure 4**). However, the chlorophyll concentration was significantly influenced by the different N fertilizer treatments (short rain season: p < 0.001, long rain season: p < 0.001) and maize growth stages (short rain season: p < 0.01), long rain season: p < 0.001) only. The interaction effects of N fertilizer treatments and maize growth stage were not significant (short rain season: p = 0.589, long rain season: p = 0.059).

Highest chlorophyll concentrations (45–61 SPAD values) were achieved at late vegetative stage (55 DAP) during the short rain season. In the short rain season, chlorophyll concentrations for all treatments significantly decreased and reached their lowest values at silking stage. During the long rain season, however, a significant increase in chlorophyll concentrations was observed at both late vegetative and silking stages. Plots treated with BSF frass fertilizer applied at 5 and 7.5 t ha^-1^, 60 and 100 kg N ha^-1^ as well as urea fertilizer applied at 100 kg N ha^-1^ achieved highest chlorophyll levels (83–93 SPAD values) at silking stage compared to other treatments which peaked at late vegetative stage.

Plots treated with BSF frass fertilizer at 7.5 t ha^-1^ produced plants with the highest chlorophyll concentrations, which were 18% (p = 0.02) and 20% (p < 0.01) higher than those achieved using an equivalent rates of SAFI in the short and long rain seasons, respectively (**Figures 4A, B**). During the long rain season, the highest chlorophyll concentration among the N fertilizer treatments was recorded from plants treated with 100 kg N ha^-1^ supplied as BSF frass fertilizer, and this was 32 and 7% (p < 0.01) higher those from plants treated with equivalent rates of SAFI and urea fertilizer, respectively.

### Effects of BSF Frass, Commercial Organic, and Urea Fertilizers on Soil Mineral N Concentration

#### Types of Fertilizers

At 0–20 cm depth, the mineral N concentration during maize growth was significantly influenced by different types of fertilizers (short rain season: p < 0.001, long rain season: p < 0.05) and maize growth stage (short rain season: p < 0.001, long rain season: p < 0.001). The interaction of fertilizer treatments and maize growth stages was significant during the short rain season only (p < 0.001) (**Table 2**). The mineral N concentration significantly increased to highest levels at the early vegetative stage (6–36 kg N ha^-1^), before decreasing to significantly minimum values at tasseling stage. Thereafter, slight changes in mineral N were observed from tasseling to maturity stage. At the early vegetative stage, the mineral N concentration of plots treated with 7.5 t ha^-1^ of BSF frass fertilizer was 4 (p < 0.01) and 6 times (p < 0.01) higher than those achieved at equivalent rates of SAFI during the short and long rain season, respectively. Likewise, plots treated with 5 t ha^-1^ of BSF frass fertilizer had 2.4 times higher (p < 0.05) mineral N concentration in soil that those treated with equivalent rate of SAFI at tasseling stage during the short rain season. Increase in mineral N concentration were observed at the maturity stage for all treatments during the long rain season whereby, plots treated with 5 t ha^-1^ of BSF frass fertilizer had 2.4 times higher mineral N concentration than where an equivalent rate of SAFI was applied.

**Table 2:** Mineral nitrogen (kg N ha^-1^) in the top 40 cm of soil treated with different types of fertilizers.

SEASON 2019A (Short rain)						SEASON 2019B (Long rain)					
Soil depth(cm)	Rate(t ha^-1^)		Days after planting								
		0	35	70	91	125	0	35	70	91	125
0–20	0	4.1 ± 1.1b	9.9 ± 2.8b	2.4 ± 0.8b	2.9 ± 2.2a	6.3 ± 0.8	39.0 ± 3.8	5.7 ± 1.0b	26.1 ± 3.1	14.4 ± 0.5	31.5 ± 0.8
	2.5BSF	4.1 ± 1.5b	8.8 ± 3.2b	5.1 ± 1.6ab	3.5 ± 2.5a	6.4 ± 1.5	39.0 ± 3.8	14.5 ± 1.9ab	63.3 ± 9.2	16.5 ± 2.1	32.4 ± 7.9
	5BSF	13.3 ± 4.7ab	28.9 ± 10ab	12.2 ± 2.7a	11.3 ± 2.4a	8.6 ± 0.6	44.8 ± 6.4	8.6 ± 2.0b	107.4 ± 70.5	20.7 ± 3.3	51.6 ± 21.9
	7.5BSF	15.7 ± 2.3a	35.7 ± 5.8a	8.1 ± 3.5ab	9.5 ± 0.5a	7.6 ± 1.1	40 ± 3.3	38.3 ± 12.9a	37 ± 6.5	19.5 ± 0.7	44.6 ± 14.1
	2.5SAFI	2.8 ± 0.6b	6.0 ± 1.2b	1.6 ± 0.1b	5.8 ± 0.6a	8.8 ± 2.6	38.2 ± 8.3	3.9 ± 0.1b	26.2 ± 4.5	16.4 ± 1.1	40.7 ± 16.6
	5SAFI	3.4 ± 1.4b	7.3 ± 3.0b	5.0 ± 2.1ab	3.7 ± 2.1a	5.9 ± 1.2	39.1 ± 4.4	3.8 ± 0.03b	18.8 ± 3.2	17.2 ± 0.4	21.2 ± 6.2
	7.5SAFI	3.7 ± 1.1b	8.0 ± 2.5b	5.4 ± 0.8ab	4.8 ± 1.6a	6.9 ± 0.5	24.8 ± 2.3	6.8 ± 2.8b	27.8 ± 8.2	18.2 ± 2.3	41.9 ± 14
		**	**	*	*	ns	ns	**	ns	ns	ns
20–40	0	3.4 ± 1.3	7.8 ± 2.9	6.4 ± 1.7	5.9 ± 0.8	3.6 ± 0.8ab	16.0 ± 6.3	19.1± 8.7b	35.9± 15	11.6 ± 3.6	12.3 ± 1.2
	2.5BSF	2.1 ± 0.7	4.1 ± 1.3	9.0 ± 2.0	6.6 ± 1.0	3.5 ± 1.3ab	24.7 ± 4.4	46.1 ± 0.5b	29.7 ± 16.9	14 ± 4.3	11.3 ± 2.5
	5BSF	3.2 ± 0.3	6.2 ± 0.5	12.2 ± 2.0	7.8 ± 1.2	5.3 ± 0.9ab	32.3 ± 21.5	41.5 ± 12b	18.6 ± 5.0	10.6 ± 0.3	17.9± 4.1
	7.5BSF	3.7 ± 0.6	7.4 ± 1.0	10.8 ± 1.1	6.7 ± 0.4	8.2 ± 2.2a	29.7 ± 13.4	119 ± 18.5a	43.3 ± 6.4	18 ± 3.9	13.4 ± 5.8
	2.5SAFI	1.2 ± 0.4	2.4 ± 0.7	9.0 ± 1.8	5.0 ± 1.1	4.8 ± 0.4ab	22.7 ± 4.5	17.2 ± 7.1b	37.7 ± 1.1	10.1 ± 3.5	6.9 ± 0.4
	5SAFI	1.6 ± 0.1	3.3 ± 0.2	9.5 ± 1.7	5.3 ± 0.6	3.4 ± 0.7ab	15.3 ± 3.5	11 ± 1.5b	31.9 ± 9.1	18.4 ± 0.4	20 ± 2.5
	7.5SAFI	2.6 ± 0.5	5.0 ± 1.0	8.5 ± 2.0	6.0 ± 0.8	2.3 ± 0.4b	19.1 ± 2.5	32 ± 11.5b	58.9 ± 24.9	10 ± 4.2	24.5 ± 8.5
		ns	ns	ns	ns	*	ns	***	ns	ns	ns

***p < 0.001, **p < 0.01, *p < 0.05, ns, not significant; 2.5, 5, and 7.5, application rates of organic fertilizers at 2.5, 5, and 7.5 t ha^-1^; BSF, black soldier fly frass fertilizer; SAFI, commercial organic fertilizer; 0, control treatment.

At soil depths of 20–40 cm, the mineral N concentration was also significantly influenced by the different types of fertilizers (short rain season: p < 0.001, long rain season: p < 0.001) and crop growth stage (short rain season: p < 0.001, long rain season: p < 0.001). The interaction effect of fertilizer types and maize growth stages was significant (p < 0.001) during the long rain season only. Peak mineral N concentration during the short rain season was achieved at tasseling stage (70 DAP) with the highest recorded in plots treated with 7.5 t ha^-1^ of BSF frass fertilizer. The same treatment had significantly (p < 0.05) higher mineral N concentration at maturity stage compared to plots treated with 7.5 t ha^-1^ of SAFI. During the long rain season, peak mineral N concentration was attained at early vegetative and silking stages for BSF frass fertilizer and SAFI treatments, respectively. Soil treated with 7.5 t ha^-1^ BSF frass fertilizer had significantly (p < 0.001) higher mineral N concentration than other treatments at the early vegetative stage.

#### Nitrogen Fertilizer Treatments

The mineral N concentration in the soil layer 0–20 cm varied significantly at different maize growth stages during the long rain season only (p < 0.05) (**Table 3**). The mineral N significantly increased to peak levels between vegetative and silking stages before decreasing gradually to the end of the short rain season. However, no significant differences in mineral N concentration were observed between the treatments. During the long rain season, mineral N significantly decreased from initial values to lowest levels at early vegetative stage. Plots treated with 60 kg N ha^-1^ supplied as BSF frass fertilizer and urea fertilizer had the highest mineral N levels at tasseling and silking stages, respectively.

**Table 3:** Mineral nitrogen (kg N ha^-1^) in the top 40 cm of soil treated with different N fertilizers.

SEASON 2019A (Short rain)						SEASON 2019B (Long rain)					
**Soil depth(cm)**	**Rate(kg N ha^-1^)**		**Days after planting**								
		**0**	**35**	**70**	**91**	**125**	**0**	**35**	**70**	**91**	**125**
0–20	0	4.1 ± 1.1	9.9 ± 2.8	2.4 ± 0.8	2.9 ± 2.2	6.3 ± 0.8	39.0 ± 3.8	5.7 ± 1.0	26.1 ± 3.1a	14.4 ± 0.5	31.5 ± 0.8
	30N BSF	3.2 ± 1.6	6.9 ± 3.6	5.7 ± 1.4	5.4 ± 2.4	6.7 ± 1.3	42.1 ± 4.2	11.4 ± 5.2	37 ± 8.7a	19.9 ± 3.3	23.5 ± 16.5
	60N BSF	2.2 ± 0.1	4.8 ± 0.2	3.4 ± 1.5	8.1 ± 1.0	6.3 ± 1.0	43.2 ± 7.7	23 ± 9.9	63.0 ± 14.5a	15.4 ± 1.4	23.4 ± 13.1
	100N BSF	4.2 ± 1.1	9.3 ± 3.0	4.2 ± 1.6	2.5 ± 2.2	6.7 ± 0.6	36.2 ± 7.9	6.4 ± 3.1	50.1 ± 6.1a	27.5 ± 4.7	54.4 ± 7.1
	30N SAFI	1.9 ± 1.1	4.4 ± 2.6	5.1 ± 1.9	6.0 ± 0.9	5.8 ± 1.0	38.1 ± 4.9	4.0 ± 0.1	28.9 ± 8.5a	17.9 ± 2.0	36.6 ± 4.5
	60N SAFI	1.8 ± 0.2	4.0 ± 0.2	11.3 ± 5.7	0.82 ± 0.1	5.4 ± 1.8	69.6 ± 10.2	3.9 ± 0.01	62.7 ± 15.0a	12.6 ± 5.6	59.2 ± 10.3
	100N SAFI	5.7 ± 1.7	12.9 ± 3.4	7.2 ± 1.0	4.8 ± 1.8	5.9 ± 1.6	46.5 ± 23.3	5.4 ± 1.6	47.1 ± 7.1a	16 ± 1.1	31.6 ± 1.8
	30N UREA	1.7 ± 0.8	3.7 ± 1.9	7.3 ± 1.2	5.5 ± 2.2	7.1 ± 2.1	46.2 ± 1.3	6.8 ± 1.6	23.6 ± 4.7a	14.5 ± 2.4	41.5 ± 3.5
	60N UREA	2.6 ± 1.3	5.9 ± 3.1	6.1 ± 0.7	8.1 ± 0.7	7.5 ± 1.2	25.4 ± 5.0	5.2 ± 1.6	45.1 ± 8.3a	32 ± 15.5	49.5 ± 23
	100N UREA	6.1 ± 0.5	13.3 ± 0.7	6.6 ± 1.1	8.2 ± 3.9	13.0 ± 3.9	39.9 ± 11.4	13.3 ± 2.9	51.1 ± 7.8a	24.4 ± 5.5	62.6 ± 7.6
		ns	ns	ns	ns	ns	ns	ns	*	ns	ns
20–40	0	3.4 ± 1.3	7.8 ± 2.9	6.4 ± 1.7	5.9 ± 0.8ab	3.6 ± 0.8a	16.0 ± 6.3	19.1 ± 9b	35.9 ± 15	11.6 ± 3.6	12.3 ± 1.2ab
	30N BSF	2.0 ± 1.1	3.9 ± 2.0	11.4 ± 0.4	5.0 ± 0.4b	3.1 ± 0.4a	17.5 ± 4.2	27.9 ± 11ab	44.5 ± 16.4	15.2 ± 6.2	9.1 ± 2.6b
	60N BSF	1.2 ± 0.4	2.5 ± 0.7	6.2 ± 0.8	5.9 ± 0.5ab	3.1 ± 0.6a	18 ± 11.2	42.5 ± 19ab	37.5 ± 10.8	17.1 ± 6.8	9.1 ± 2.0b
	100N BSF	2.1 ± 0.1	4.1 ± 0.1	7.8 ± 3.3	5.3 ± 0.2ab	4.6 ± 0.6a	19 ± 4.4	34.5 ± 11ab	49.6 ± 8.1	11.0 ± 2.7	15.9 ± 5.0ab
	30N SAFI	1.2 ± 0.4	2.5 ± 0.7	7.5 ± 1.6	4.5 ± 0.3b	3.0 ± 0.2a	32.5 ± 12.0	16.5 ± 8.3b	65.6 ± 1.5	10.2 ± 0.4	7.5 ± 1.0b
	60N SAFI	0.8 ± 0.4	1.8 ± 0.7	7.4 ± 1.6	5.1 ± 0.2ab	3.6 ± 0.5a	38.9 ± 15.4	32.4 ± 14ab	42 ± 8.3	11.1 ± 6.8	9.4 ± 1.6b
	100N SAFI	2.4 ± 0.9	4.6 ± 1.7	7.3 ± 2.8	6.4 ± 0.6ab	2.8 ± 1.3a	13.4 ± 6.0	8.2 ± 2.1b	35.9 ± 11.2	12.4 ± 4.6	6.5 ± 0.1b
	30N UREA	2.1 ± 0.9	4.0 ± 1.6	12.1 ± 2.5	5.1 ± 0.1ab	5.9 ± 0.8a	9.6 ± 2.8	12.4 ± 2.1b	36.8 ± 13.8	16.1 ± 6.4	20.5 ± 4.1ab
	60N UREA	1.1 ± 0.3	2.3 ± 0.5	9.9 ± 2.4	7.5 ± 1.3ab	3.9 ± 0.5a	19.7 ± 8.7	89.5 ± 22ab	48.1 ± 14	12.7 ± 3.5	26.1 ± 5.5a
	100N UREA	2.4 ± 1.0	4.6 ± 1.8	15.5 ± 5.2	8.9 ± 1.6a	6.2 ± 0.5a	35.6 ± 6.5	149.3 ± 69a	108.2 ± 41	26.2 ± 6.9	20.6 ± 1.5ab
		ns	ns	ns	*	*	ns	*	ns	ns	**

*p < 0.05, ns, not significant; 30N, 60N, and 100N, application rates of fertilizers at rates equivalent to 30, 60, and 100 kg N ha^-1^ supplemented with 60 kg P ha^-1^ and 50 kg K ha^-1^; 0, control treatment; BSF, black soldier fly frass fertilizer; SAFI, commercial organic fertilizer.

In the soil layer 20–40 cm, mineral N concentration in soil was significantly affected by N fertilizer treatments (short rain season: p < 0.001, long rain season: p < 0.001) and maize growth stage (short rain season: p < 0.001, long rain season: p < 0.001). The interaction of N fertilizer treatments and maize growth stage was significant during the long rain season only (p < 0.01). Mineral N concentration significantly increased to peak levels (6.2–15.5 kg N ha^-1^) at tasseling stage, before gradually decreasing toward the end of the short rain season. Plots treated with 100 kg N ha^-1^ supplied as urea had significantly (p < 0.05) higher mineral N concentration at silking stage than where 30 kg N ha^-1^ from SAFI and 60 kg N ha^-1^ supplied as BSF frass fertilizer were applied. Similarly, plots treated with 100 kg N ha^-1^ supplied as urea achieved significantly (p < 0.05) higher mineral N concentration than equivalent rate of SAFI at early vegetative stage during the long rain season. At maturity stage (125 DAP) of the same season, plots treated with 60 kg N ha^-1^ applied as urea fertilizer had significantly (p < 0.01) higher mineral N concentration than BSF frass and SAFI treatments, except for plots where 100 kg N ha^-1^ of BSF frass fertilizer was applied.

### Effects of BSF Frass, Commercial Organic, and Urea Fertilizers on N, P, and K Uptake

#### Types of Fertilizers

**Figure 5:**
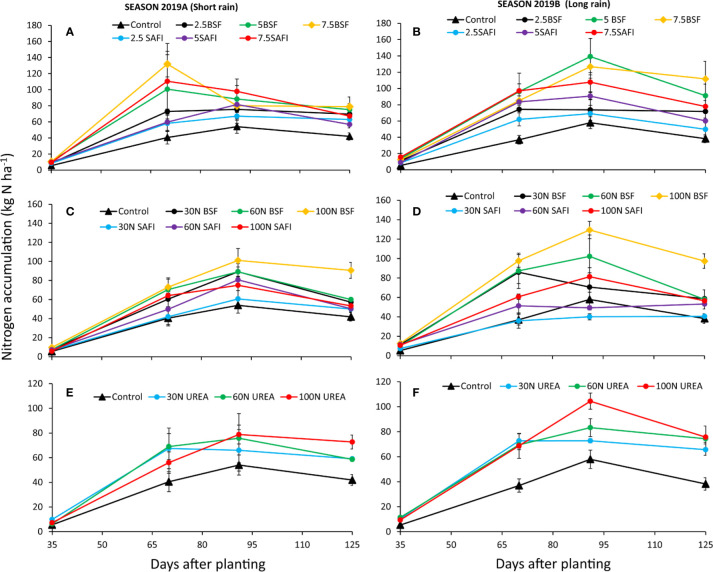
Effect of black soldier fly (BSF) frass fertilizer **(A–D)**, commercial organic (SAFI) fertilizer **(A–D)**, and commercial mineral fertilizer **(E, F)** on nitrogen uptake by maize.

The quantity of nitrogen accumulated in maize biomass was significantly influenced by the different types of fertilizers (short rain season: p < 0.001, long rain season: p < 0.001) and maize growth stages (short rain season: p < 0.001, long rain season: p < 0.001) (**Figures 5A, B**). The interaction of fertilizer types and growth stages was significant only during the long rain season (p < 0.05). Nitrogen uptake significantly increased from 9–16 kg N ha^-1^ at early vegetative stage (35 DAP) to highest values (40–139 kg N ha^-1^) at tasseling (70 DAP) and silking stages (91 DAP). Thereafter, N uptake levels decreased gradually to maturity stage. Maize grown using 7.5 t ha^-1^ of BSF frass fertilizer accumulated the highest N levels at tasseling stage during the long rain season experiments, while maize grown on plots treated with 5 t ha^-1^ of BSF frass fertilizer accumulated significantly (p < 0.01) higher N at the silking stage than those where 2.5 t ha^-1^ of either BSF frass or SAFI was applied during the long rain season. At maturity stage, the N accumulated (38–112 kg ha^-1^) varied significantly (short rain season: p < 0.05, long rain reason: p < 0.01) among the treatments. The N accumulated in maize treated with 7.5 t ha^-1^ of BSF frass fertilizer was 17 and 44% higher than N accumulated in maize treated with equivalent rates of the SAFI by during the short and long rain seasons, respectively, but the differences were not statistically significant

There were significant differences in the quantity of P accumulated in maize biomass at harvesting stage among the different types of fertilizers during the short rain (p < 0.05) and long rain season (p < 0.01) (**Table 4**). Total P accumulated in maize treated with 5 and 7.5 t ha^-1^ of BSF frass fertilizer were significantly higher than those of the control treatment in both seasons. The total P accumulated in plots treated with 7.5 t ha^-1^ of BSF frass fertilizer was 15 and 27% higher than the P accumulated by maize grown using similar rates of SAFI during the short and long rain seasons, respectively.

**Table 4:** Effects of black soldier fly (BSF) frass fertilizer, commercial organic and mineral (urea) fertilizers on maize stover yields, and phosphorus and potassium uptake at maturity.

Fertilizer treatments	SEASON 2019A (Short rain)			SEASON 2019B (Long rain)				
	Stover yield(t ha^-1^)	Nutrients uptake (kg ha^-1^)		Stover yield(t ha^-1^)	Nutrients uptake (kg ha^-1^)			
		Phosphorus	Potassium		Phosphorus		Potassium	
**Types of fertilizers**								
Control	5.9 ± 0.78	4.2 ± 0.22b	77.6 ± 10.3b	5.8 ± 1.2		5.4 ± 1.3b		63.6 ± 13.7b
2.5 BSF	8.4 ± 0.63	12.7 ± 2.1a	128.5 ± 25ab	8.1 ± 0.6		12.7 ± 1.3ab		122.0 ± 14.8ab
5 BSF	9.6 ± 0.76	12.9 ± 1.9a	160.5 ± 15.3ab	9.8 ± 2.4		16.3 ± 2.5a		163.8 ± 34.5ab
7. 5BSF	7.9 ± 1.05	13.3 ± 1.2a	118.9 ± 10.9ab	11.5 ± 2.3		18.6 ± 2.8a		173.4 ± 37.0ab
2.5 SAFI	8.5 ± 0.70	10.7 ± 2.2ab	97.4 ± 18.7ab	7.2 ± 1.9		10.2 ± 1.5ab		123.5 ± 32.5ab
5 SAFI	6.4 ± 0.44	11.1 ± 1.2ab	144.0 ± 22.8ab	7.4 ± 0.6		11.8 ± 1.8ab		110.1 ± 21.6ab
7.5 SAFI	8.8 ± 1.49	11.6 ± 2.1ab	171.2 ± 10.6a	10.6 ± 1.8		14.6 ± 1.8ab		212.5 ± 33.5a
Fertilizer	ns	*	*	ns		**		*
**Nitrogen fertilizer treatments**								
30N BSF	6.8 ± 1.78	10.6 ± 0.9ab	104.9 ± 20.9ab	7.7 ± 0.32abc		11.7 ± 0.6abc		125.1 ± 21.6ab
60N BSF	8.3 ± 0.83	13.6 ± 1.8ab	109.4 ± 12.8ab	8.9 ± 0.26ab		14.6 ± 2.4ab		115.5 ± 1.8ab
100N BSF	9.6 ± 1.41	16.4 ± 2.0a	179.0 ± 37.5a	10.3 ± 0.10a		17.4 ± 1.0a		189.2 ± 30.9a
30N SAFI	6.1 ± 0.60	8.7 ± 0.6ab	83.3 ± 14.1ab	5.0 ± 0.34c		7.2 ± 0.9bc		69.0 ± 13.5b
60N SAFI	7.6 ± 0.97	7.2 ± 1.1bc	98.0 ± 19.9ab	7.3 ± 0.27abc		8.1 ± 0.4bc		94.1 ± 16.8b
100N SAFI	7.7 ± 0.71	10.8 ± 1.3ab	119.0 ± 4.4ab	8.0 ± 0.20abc		11.8 ± 1.3abc		124.7 ± 2.7ab
30N UREA	9.0 ± 1.23	10.6 ± 0.8ab	143.2 ± 28.5ab	7.1± 0.4babc		12.7 ± 1.1abc		113.3 ± 7.3ab
60N UREA	6.4 ± 0.89	10.0 ± 2.6ab	91.6 ± 11.3ab	9.1± 0.04ab		12.6 ± 3.3abc		129.5 ± 5.5ab
100N UREA	8.1 ± 0.54	12.6 ± 2.3ab	121.4 ± 11.1ab	7.7± 0.42abc		13.3 ± 2.9abc		118.3 ± 13.6ab
Control	5.9 ± 0.78	4.2 ± 0.2c	77.6 ± 10.3b	5.8 ± 1.2bc		5.4 ± 1.3c		63.6 ± 13.7b
	ns	**	*	**		**		***

***p < 0.001, **p < 0.01, *p < 0.05, ns, not significant; 2.5, 5, and 7.5, application rates of organic fertilizers at 2.5, 5, and 7.5 t ha^-1^; 30N, 60N, and 100N, application rates of fertilizers at rates equivalent to 30, 60, and 100 kg N ha^-1^ supplemented with 60 kg P ha^-1^ and 50 kg K ha^-1^; BSF, black soldier fly frass fertilizer; SAFI, commercial organic fertilizer.

Accumulated K also varied significantly in both seasons due to different types of fertilizers (short rain season: p < 0.05, long rain season: p < 0.05) (**Table 4**). Maize grown using 7.5 t ha^-1^ of SAFI accumulated significantly higher K contents in both seasons than maize grown in the control treatments. On the other hand, the total K accumulated in plots treated with 7.5 t ha^-1^ was 44 and 23% higher than that accumulated using equivalent rates of BSF frass fertilizer during the short and long rain seasons, respectively.

#### Nitrogen Fertilizer Treatments

The different N fertilizer treatments significantly (short rain season: p < 0.001, long rain season: p < 0.001) influenced the N uptake in maize biomass (**Figures 5C–F**). Like with types of fertilizers (section 3.4.1), N uptake also varied significantly at different maize growth stages (short rain season: p < 0.001, long rain season: p < 0.001). The interaction effect of N fertilizer treatments and growth stages was significant during the long rain season only (p < 0.001). The N accumulation at early vegetative stage (5–13 kg N ha^-1^) significantly (p < 0.001) increased to highest values at tasseling and silking stages (40–129 kg N ha^-1^). A significant (p < 0.001) decrease in N uptake was observed at maturity stage, except for plots treated with 30 and 60 kg N ha^-1^ supplied using SAFI during the long rain season. Plots treated with 60 and 100 kg N ha^-1^ supplied as BSF frass fertilizer accumulated higher N levels at tasseling (p < 0.001) and silking stages (p < 0.001) than equivalent rates of commercial organic and urea fertilizers.

The N accumulated in maize treated with 100 kg N ha^-1^ of BSF frass fertilizer at tasseling stage of the long rain season was significantly (p < 0.001) higher than those of maize grown using 30 and 60 kg N ha^-1^ supplied using SAFI. Similarly, application of 100 kg N ha^-1^ as BSF frass fertilizer caused significantly (p < 0.001) higher N uptake at silking stage of the same season compared to where 30 kg N ha^-1^ of all fertilizers, and 60 kg N ha^-1^ of SAFI were applied. Application of 100 kg N ha^-1^ supplied as BSF frass fertilizer caused significantly (short rain season: p < 0.001, long rain season: p < 0.001) higher N accumulation than other treatments at harvesting stage, except for maize grown using 100 kg N ha^-1^ of urea fertilizer in both seasons, and 60 kg N ha^-1^ of urea during the long rain season.

Phosphorus accumulated in maize biomass at maturity stage (4–17 kg P ha^-1^) was significantly influenced by different N fertilizer treatments (short rain season: p < 0.01, long rain season: p < 0.01) (**Table 4**). Maize grown using 100 kg N ha^-1^ supplied as BSF frass fertilizer accumulated significantly higher P levels than those grown using 60 kg N ha^-1^ of SAFI and control treatment in both seasons, and 30 kg N ha^-1^ of SAFI during the long rain season. Similarly, application of 100 kg N ha^-1^ supplied as BSF frass fertilizer caused significantly higher K accumulation than the control treatment in both seasons (short rain season: p < 0.05, long rain season: p < 0.001), and where 30 and 60 kg N ha^-1^ of SAFI were applied during the long rain season.

### Effects of BSF Frass, Commercial Organic, and Urea Fertilizers on Maize Grain and Stover Yields

#### Types of Fertilizers

**Figure 6:**
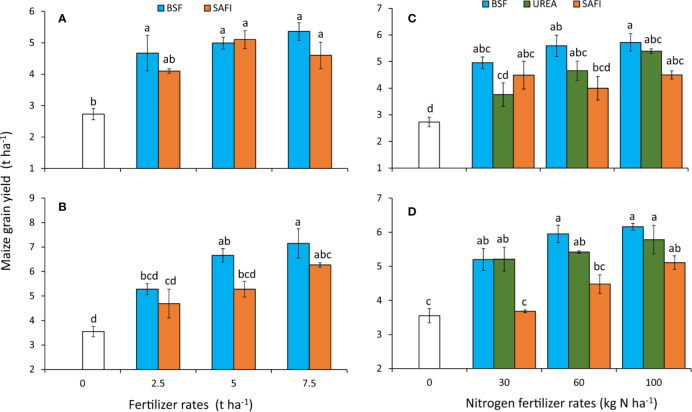
Effect of fertilizer types **(A, B)** and nitrogen rates **(C, D)** on maize grain yields during the short **(A, C)** and long rain season **(B, D)** experiments.

The different types of fertilizers caused significant (short rain season: p < 0.001, long rain season: p < 0.001) differences in maize grain yields during experiments (**Figures 6A, B**). Application of BSF frass fertilizers increased grain yields by 71%96% during the short rain season and 49%–101% during long rain compared to the control. On the other hand, grain yields increased by 50%–87% during the short rains and 32%–77% during the long rains season due to SAFI. For SAFI, significant (p < 0.001) increases in grain yield above the control were only achieved at rates above of 5 t ha^-1^ and above during the short rain season, and at 7.5 t ha^-1^ during the long rain season. Maize grain yields did not vary significantly at equivalent rates of the commercial organic and BSF frass fertilizers.

Application of BSF frass fertilizer at 7.5 t ha^-1^ produced the highest maize grain yields during the short (5.4 t ha^-1^) and long rain season (7.2 t ha^-1^) which were % and 14% higher than the highest grain yields obtained using SAFI during the short and long rain seasons, respectively, but not significantly different. Grain yield from plots treated with 7.5 t ha^-1^ of BSF frass fertilizer was significantly (p < 0.001) higher than that where 2.5 t ha^-1^ of SAFI were applied during the short rain season. Similarly, grain yield from maize grown using 7.5 t ha^-1^ of BSF frass compost was significantly (p < 0.001) higher than those of other treatments during the long rain season, except for maize treated with 5 t ha^-1^ BSF frass fertilizer and 7.5 t ha^-1^ of SAFI.

The different sole BSF frass and SAFI treatments did not significantly influence maize stover yields during experiments (short rain season: p = 0.12, long rain season: p = 0.26) (**Table 4**). However, plots treated with BSF frass fertilizer had higher stover yields than those treated with SAFI during the long rain season.

#### Nitrogen Fertilizer Treatments

The grain yields also varied significantly (short rain season: p < 0.001, long rain season: p < 0.001) among the different N fertilizer treatments tested (**Figures 6C, D**). All plots treated with BSF frass fertilizer produced significantly (p < 0.001) higher grain yields than the control. Grain yields from plots treated with 100 kg N ha^-1^ of BSF frass fertilizer were significantly (p < 0.001) higher than those where 30 kg N ha^-1^ of urea and 60 kg N ha^-1^ of SAFI were applied during the short rain season (**Figure 6C**). Similarly, plots treated with 60 and 100 kg N ha^-1^ of BSF frass fertilizer as well 100 kg N ha^-1^ of urea produced significantly (p < 0.001) higher maize grain yields than where 30 and 60 kg N ha^-1^ of SAFI were applied during the long rain season (**Figure 6D**).

Maize treated with 100 kg N ha^-1^ applied as BSF frass fertilizer produced the highest grain yield (5.7 t ha^-1^) during the short rain season which was 6% and 27% higher than yields obtained using equivalent rates of urea and SAFI, respectively. Likewise, grain yield from maize treated with 100 kg N ha^-1^ applied as BSF frass fertilizer (6.2 t ha^-1^) was 7% and 21% higher than those obtained using equivalent rates of urea and SAFI, respectively, during the long rain season. The stover yields varied significantly (p < 0.01) during the long rain season only (**Table 4**). Plots treated with 100 kg N ha^-1^ supplied as BSF frass fertilizer produced significantly (p < 0.01) higher stover maize yields than the control treatment and where 30 kg N ha^-1^ supplied using SAFI was applied.

### Nitrogen Fertilizer Equivalences of BSF Frass and Commercial Organic Fertilizers

The highest maize grain yields from organic fertilizer treatments were 5.7 and 4.5 t ha^-1^ for BSF frass fertilizer and SAFI, respectively, compared to 5.4 t ha^-1^ from urea treatment (all applied at 100 kg N ha^-1^) in the short rain season. This gave NFE values of 102% for BSF frass- fertilizer and 51% for SAFI (**Figure 2A**). During the long rain season, the highest maize grain yields were 6.2 and 5.1 t ha^-1^ for BSF frass fertilizer and SAFI, respectively, compared to 5.8 t ha^-1^ from urea treatment (all applied at 100 kg N ha^-1^). This gave NFE values of 114 and 35% for the BSF frass fertilizer and SAFI, respectively (**Figure 2B**). Over the two seasons, the average NFE value of BSF frass fertilizer was (108%) while that of SAFI (43%), 2.5 times lower than that of BSF frass fertilizer.

### Effects of BSF Frass, Commercial Organic, and Urea Fertilizers on Nitrogen Use Efficiencies

#### Agronomic Nitrogen Efficiency (AE_N_)

The various types of fertilizers (short rain season: p < 0.01, long rain season: p < 0.05) and N fertilizers (short rain season: p < 0.01, long rain season: p < 0.001) caused significant differences in AE_N_ of maize during experiments (**Table 5**). Application of 2.5 t ha^-1^ of BSF frass fertilizer increased the AE_N_ of maize by 2.4 and 2.2 times compared to equivalent rate of SAFI during the short and long rain season, respectively. Further, maize grown using 2.5 t ha^-1^ of BSF frass fertilizer achieved significantly higher AE_N_ than other treatments, except for plots treated with 5 and 7.5 t ha^-1^ of the same fertilizer and 2.5 t ha^-1^ of SAFI during the long rain season.

**Table 5:** Effects of black soldier fly (BSF) frass fertilizer, commercial organic, and mineral (urea) fertilizers on nitrogen use efficiency of maize.

Fertilizer treatments	SEASON 2019A (Short rain)				SEASON 2019B (Long rain)			
	AE_N_(kg kg^-1^ N)	ANR_N_(%)	APE_N_(kg kg^-1^ N)	NHI(%)	AE_N_(kg kg^-1^ N)	ANR_N_(%)	APE_N_(kg kg^-1^ N)	NHI(%)
**Types of fertilizers**								
2.5 BSF	43.2 ± 10.8a	52.8 ± 2.4a	81.2 ± 19.6b	52.9 ± 3.3ab	33.0 ± 4.2a	63.8 ± 15.0a	55.5 ± 9.2	55.4 ± 1.9
5 BSF	18.5 ± 1.8b	31.7 ± 7.0ab	62.5 ± 9.6b	51.3 ± 5.4ab	29.7 ± 2.7ab	50.3 ± 13.5a	69.9 ± 23.2	60.9 ± 4.6
7.5 BSF	16.7 ± 1.8b	23.5 ± 7.7b	80.9 ± 15.1b	53.4 ± 1.4ab	22.9 ± 3.8ab	46.7 ± 13.7a	56.2 ± 12.5	52.4 ± 6.1
2.5 SAFI	18.4 ± 1.0b	28.0 ± 1.9b	66.5 ± 7.6b	42.6 ± 4.0b	15.3 ± 7.8ab	15.5 ± 11.1a	35.7 ± 13.8	54.4 ± 9.4
5 SAFI	15.8 ± 1.9b	9.9 ± 2.8b	177.7 ± 36.9a	67.5 ± 5.6a	11.6 ± 2.1b	14.6 ± 1.1a	81.1 ± 17.5	65.7 ± 5.0
7.5 SAFI	18.3 ± 1.9b	11.3 ± 1.5b	72.3 ± 6.4b	42.7 ± 2.4b	12.1 ± 0.4b	17.6 ± 3.3a	74.6 ± 15.1	51.9 ± 6.9
Control	na	na	na	55.2 ± 2.0ab	na	na	na	51.6 ± 5.8
	**	***	**	**	*	*	ns	ns
**Nitrogen fertilizer treatments**								
30N BSF	74.4 ± 7.1a	50.9 ± 9.5a	153.4 ± 22.3ab	54.3 ± 5.9	55.0 ± 10.7a	68.2 ± 3.4ab	79.9 ± 13.8	55.4 ± 7.0
60N BSF	47.7 ± 6.7ab	30.2 ± 3.7ab	165.5 ± 34.8ab	52.2 ± 7.0	40.0 ± 4.3ab	32.9 ± 6.3bcd	82.4 ± 10.4	58.6 ± 3.3
100N BSF	29.9 ± 3.2b	48.7 ± 8.3a	64.6 ± 12.0b	62.8 ± 2.2	26.1 ± 1.0abc	59.2 ± 7.5abc	45.2 ± 4.5	62.9 ± 0.8
30N SAFI	58.8 ± 17.4ab	27.0 ± 10.7ab	234.7 ± 43.3a	50.3 ± 2.0	4.4 ± 1.3c	7.5 ± 2.7d	98.4 ± 58.2	64.6 ± 1.5
60N SAFI	21.3 ± 7.4b	14.0 ± 5.3b	156.2 ± 14.7ab	56.0 ± 1.6	15.6 ± 4.6bc	25.1 ± 8.7bcd	66.7 ± 18.0	58.9 ± 5.7
100N SAFI	17.8 ± 1.5b	11.4 ± 4.0b	182.0 ± 38.8ab	56.4 ± 7.2	15.7 ± 2.0bc	18.2 ± 4.2cd	91.1 ± 11.4	57.0 ± 6.9
30N UREA	34.4 ± 14.8ab	57.3 ± 1.5a	85.6 ± 2.3b	54.4 ± 8.1	55.3 ± 11.8a	91.4 ± 15.2a	60.3 ± 9.5	67.4 ± 3.3
60N UREA	32.2 ± 6.0ab	27.7 ± 2.7ab	122.6 ± 35.0ab	56.8 ± 6.8	31.3 ± 0.6abc	60.3 ± 4.8abc	52.5 ± 3.8	51.8 ± 2.4
100N UREA	26.7 ± 0.9b	30.8 ± 5.7ab	95.8 ± 24.6ab	58.4 ± 1.9	22.4 ± 4.2bc	37.4 ± 9.0bcd	61.7 ± 4.9	60.6 ± 4.4
Control	na	na	na	55.2 ± 2.0	na	na	na	51.6 ± 5.9
	**	***	*	ns	***	***	ns	ns

***p < 0.001, **p < 0.01, *p < 0.05, ns, not significant; AE_N_, agronomic nitrogen efficiency; ANR_N_, apparent nutrient recovery; APE_N_, agro-physiological nitrogen efficiency; NHI, nitrogen harvest index; 2.5, 5, and 7.5, application rates of organic fertilizers at 2.5, 5 and 7.5 t ha^-1^; 30N, 60N, and 100N, application rates of fertilizers at rates equivalent to 30, 60, and 100 kg N ha^-1^ supplemented with 60 kg P ha^-1^ and 50 kg K ha^-1^; BSF, black soldier fly frass fertilizer; SAFI, commercial organic fertilizer; na, not applicable.

During the short rain season, the AE_N_ of maize treated with 30 kg N ha^-1^ of BSF frass fertilizer was significantly (p < 0.01) higher than those of maize treated with 100 kg N ha^-1^ of all fertilizers, and 60 kg N ha^-1^ of SAFI. Also, the AE_N_ in plots treated with 30 kg N ha^-1^ of BSF frass fertilizer was significantly higher than those achieved by all SAFI treatments and 100 kg N ha^-1^ of urea during the long rain season.

#### Apparent Nitrogen Recovery (ANR_N_)

The ANR_N_ of maize was significantly influenced by the different types of fertilizers (short rain season: p < 0.001, long rain season: p < 0.05) and N rates (short rain season: p < 0.001, long rain season: p < 0.001) (**Table 5**). Maize treated with 2.5 t ha^-1^ of BSF frass fertilizer had significantly (p < 0.001) higher ANR_N_ values than other treatments, except for plots where 5 t ha^-1^ of the same fertilizer were applied during the short rain season. The ANR_N_ of maize treated with 2.5 t ha^-1^ of BSF frass fertilizer was 1.9 and 4.1 times higher than that achieved by equivalent rate of SAFI during the short and long rain season, respectively.

Maize treated with 30 kg N ha^-1^ supplied as urea achieved significantly (p < 0.001) higher ANR_N_ values than other treatments in the short rain season, except for those treated with 30 and 60 kg N ha^-1^ supplied using SAFI. Similarly, the ANR_N_ of maize treated with 30 kg N ha^-1^ as urea, was significantly (p < 0.001) higher than those of other treatments during the long rain season, except for maize grown using 60 kg N ha^-1^ of urea, 30 and 100 kg N ha^-1^ of BSF frass fertilizer.

#### Agro Physiological Nitrogen Efficiency (APE_N_)

The APE_N_ of maize grown using different types of fertilizers (p < 0.01) and N rates (p < 0.05) varied significantly during the short rain season only (**Table 5**). Maize treated with 5 t ha^-1^ applied as SAFI achieved significantly (p < 0.01) higher APE_N_ values than other treatments. During the long rain season, the APE_N_ of maize treated with 5 t ha^-1^ of SAFI was 16% higher than that achieved using an equivalent rate of BSF frass fertilizer.

On the other hand, the APE_N_ of maize grown in plots treated with 30 kg N ha^-1^ supplied as SAFI was significantly (p < 0.05) higher than those attained by 100 kg N ha^-1^ of BSF frass fertilizer and 30 kg N ha^-1^ of urea fertilizer during the short rain season. During the long rain season, APE_N_ values of maize treated with 30 kg N ha^-1^ of SAFI was 63 and 23% higher than those attained using equivalent rates of urea and BSF frass fertilizer, respectively.

#### Nitrogen Harvest Index

The NHI of maize grown using different types of fertilizers varied significantly (p < 0.01) during the short rain season only (**Table 5**). Maize grown on plots treated with 5 t h^-1^ of SAFI had significantly (p < 0.01) higher NHI values than those treated with 2.5 and 7.5 t ha^-1^ of the same fertilizer treatments. Also, the NHI of maize treated with 5 t ha^-1^ of SAFI was 8% higher than that achieved using equivalent rate of BSF frass fertilizer during the long rain season. On the other hand, the different N fertilizer rates did not influence the NHI of maize significantly (short rain season: p = 0.89, long rain season: p = 0.31). Maize treated with 100 kg N ha^-1^ of BSF frass fertilizer and 30 kg N ha^-1^ of urea fertilizer, had the highest NHI values during the short and long rain season, respectively.

## Discussion

### Effects of Black Soldier Fly Frass, Commercial Organic, and Urea Fertilizers on Maize Growth, Yield, and Nitrogen Use Efficiency

Globally, intensive maize production requires a lot of fertilizer inputs, which are expensive and inaccessible to resource poor farmers, especially in low to middle income countries on top of causing environmental and health challenges (Uzoh et al., 2019). Furthermore, production of mineral fertilizers is high energy consuming (Banaeian and Zangeneh, 2011) and their use in farming is increasingly being regarded as unsustainable. Therefore, sustainable management systems for intensive maize production are sought. In this paper, we investigated for the first time how black soldier fly frass fertilizers could be used in place of conventional organic and inorganic fertilizers for maize production.

Our findings revealed higher maize growth, nutrient uptake, and grain yields associated with all fertilizer treatments compared to the control (unamended soil). From the results, it can be concluded that treating the soil with fertilizer significantly improved the maize yield and quality in terms of macronutrients (nitrogen, phosphorus, and potassium). This is consistent with other studies which clearly demonstrated that most parts of central Kenya have low soil fertility (Gachimbi et al., 2005; Muchena et al., 2005) and recommended regular fertilizer inputs for soil fertility improvement (Tully et al., 2015; Vanlauwe et al., 2015). Therefore, the increased maize plant height, chlorophyll concentration, and nitrogen and phosphorus uptake observed in plots treated with black soldier fly frass fertilizer compared to plots treated with the commercial organic and mineral fertilizers could be attributed to better supply and availability of nutrients from the newly introduced frass fertilizer. Furthermore, it is suggested that the high release of nutrients resulting from the high mineralization rate of black soldier fly frass fertilizer (Adin Yéton et al., 2019) and high availability of mineral nitrogen in the top 20 cm of soil might have partly contributed to better synchrony of nutrients supply for maize growth, chlorophyll formation and high yields. According to Tittonell et al. (2008b), increasing uptake of phosphorus in the study might have been partly responsible for high nitrogen accumulation observed in maize grown in plots treated with black soldier fly frass fertilizer. This can be attributed to the role of phosphorus in energy transfer as a component of adenosine phosphates (Fageria, 2001). Although, potassium has been equally reported to be important in nitrogen absorption and metabolism as well as plant growth (Fageria, 2001), it was taken up in sufficient quantities across all treatments.

The observations described above are in line with the report by Kagata and Ohgushi (2012), who also demonstrated that frass fertilizers is of good quality and capable of improving soil nutrient availability, and growth and yield of *Brassica rapa* L. var. rapa. Additional benefits of insect frass fertilizer on soil health for improved drought and salt tolerance, disease suppression, higher crop growth and yield have also been documented by Choi and Hassanzadeh (2019) and Houben et al. (2020).

Remarkably, higher apparent nitrogen recovery and agronomic nitrogen use efficiency values were observed in maize harvested from plots treated with black soldier fly frass fertilizer compared to those from plots treated with commercial fertilizers. The highest values of agronomic and agro-physiological nitrogen use efficiencies as well as nitrogen harvest indices observed at lower application rates of BSF frass fertilizer (2.5 t ha^-1^ and 30 kg N ha^-1^) indicate sufficiency in nitrogen supply for maize growth. Interestingly, one of the major factors limiting the use of organic fertilizers is associated with the high rates of application required (≥ 5 t ha^-1^) (Mucheru-Muna et al., 2014; Musyoka et al., 2017). Therefore, our findings would encourage a shift in attitude toward embracing the use of black soldier fly frass fertilizer. Nonetheless, further studies to determine the economically optimum rates of black soldier fly frass fertilizers for maize production are warranted.

### Nitrogen Fertilizer Equivalence of Black Soldier Fly Frass and Commercial Organic Fertilizers

The nitrogen fertilizer equivalence values obtained in this study are comparable to those reported by Kimetu et al. (2004) and Murwira et al. (2003), which ranged between 50 and 118% for other organic fertilizers (calliandra, senna and tithonia). The results obtained indicate that 102–114 kg N ha^-1^ of urea would be required to produce the same maize yield (5.7 t ha^-1^) as obtained with 100 kg N ha^-1^ for black soldier fly frass fertilizer. The nitrogen fertilizer equivalence values of black soldier fly frass fertilizer in this study are higher comparable to those reported in previous studies for different organic fertilizers (Kimetu et al., 2004; Bowden et al., 2007; Lalor et al., 2011; Cavalli et al., 2016; van Middelkoop and Holshof, 2017; De Notaris et al., 2018). Our findings demonstrate that black soldier fly frass fertilizer has potential to increase crop productivity in most developing countries where mineral fertilizer use is still limited by high prices yet less effective due to multiple soil degradation challenges.

## Conclusions

It is clear, black soldier fly frass fertilizer performed better than commercial organic and inorganic fertilizers. The various types of fertilizers showed considerable influence on maize plant height, chlorophyll concentrations and macronutrients uptake during the short and long rain cropping seasons. However, maize yield and nitrogen use efficiency were improved with the application of black soldier fly frass fertilizer. High nitrogen fertilizer equivalence of black soldier fly frass fertilizer (> 100%) indicates that it can be an effective substitute for mineral nitrogen fertilizers. Based on our findings, black soldier fly frass fertilizer if applied to supply all nutrients in maize cropping system should be applied at the rate of 2.5 t ha^-1^. However, if black soldier fly frass fertilizer should be applied to supply adequate nitrogen for maize production, a rate equivalent to 30 kg N ha^-1^ is recommended. We can conclude that black soldier fly frass fertilizer can be adopted as an environmentally safe and sustainable option for increased maize production. In the long term, we anticipate that sustainable utilization of black soldier fly frass fertilizer will reduce overreliance on the expensive mineral fertilizers with deleterious effects on soil and environmental health. Future studies to determine mid and long-term effects of black soldier fly frass fertilizer on soil fertility across different agro-ecological zones and cropping systems are crucial.

## Data Availability Statement

All datasets presented in this study are included in the article/supplementary material.

## Author Contributions

Conceptualization: DB, BM, NK, DN, KF, MM, SE, and CT. Data Curation: DB, BM, NK, MM, and CT. Formal Analysis: DB, BM, NK, KF, MM, and CT. Funding Acquisition: FMK, SS, DN, MM, SE, and CT. Investigation: DB, BM, NK, KF, MM, and CT. Methodology: DB, BM, NK, KF, MM, and CT. Project Administration: BM, NK, KF, FK, SS, MM, TD, SE, and CT. Resources: BM, NK, KF, MM, SE, and CT. Software: DB, BM, NK, MM, TD, and CT. Supervision: BM, NK, KF, MM, and CT. Validation: DB, BM, NK, KF, TD, MM, and CT Visualization: DB, BM, NK, KF, MM, and CT. Writing—Original Draft Preparation: DB, BM, NK, KF, MM, and CT. Writing—Review and Editing: DB, BM, NK, DN, KF, FK, SS, TD, MM, and CT. All authors contributed to the article and approved the submitted version.

## Funding

This research was financially supported by the Canadian International Development Research Centre (IDRC) and the Australian Centre for International Agricultural Research (ACIAR) (INSFEED-Phase 2: Cultivate Grant No: 108866-001), the Netherlands Organization for Scientific Research, WOTRO Science for Global Development (NWO-WOTRO) (ILIPA–W 08.250.202), and The Rockefeller Foundation (SiPFeed-2018 FOD 009) through the International Centre of Insect Physiology and Ecology (*icipe*). We also gratefully acknowledge the ICIPE core funding provided by United Kingdom’s Foreign, Commonwealth & Development Office (FCDO); the Swedish International Development Cooperation Agency (Sida); the Swiss Agency for Development and Cooperation (SDC); the Federal Democratic Republic of Ethiopia; and the Government of the Republic of Kenya. The views expressed herein do not necessarily reflect the official opinion of the donors. The senior author, Dennis Beesigamukama, was financially supported by a German Academic Exchange Service (DAAD) In-Region Postgraduate Scholarship.

## Conflict of Interest

The authors declare that the research was conducted in the absence of any commercial or financial relationships that could be construed as a potential conflict of interest.
